# Low perfusion compartments in glioblastoma quantified by advanced magnetic resonance imaging and correlated with patient survival

**DOI:** 10.1016/j.radonc.2019.01.008

**Published:** 2019-05

**Authors:** Chao Li, Jiun-Lin Yan, Turid Torheim, Mary A. McLean, Natalie R. Boonzaier, Jingjing Zou, Yuan Huang, Jianmin Yuan, Bart R.J. van Dijken, Tomasz Matys, Florian Markowetz, Stephen J. Price

**Affiliations:** a Cambridge Brain Tumour Imaging Laboratory, Division of Neurosurgery, Department of Clinical Neuroscience, University of Cambridge, UK; b Department of Neurosurgery, Shanghai General Hospital (originally named “Shanghai First People’s Hospital”), Shanghai Jiao Tong University School of Medicine, China; c EPSRC Centre for Mathematical Imaging in Healthcare, University of Cambridge, UK; d Department of Neurosurgery, Chang Gung Memorial Hospital, Keelung, Taiwan; e Chang Gung University College of Medicine, Taoyuan, Taiwan; f Cancer Research UK Cambridge Institute, University of Cambridge, UK; g CRUK & EPSRC Cancer Imaging Centre in Cambridge and Manchester, Cambridge, UK; h Developmental Imaging and Biophysics Section, Great Ormond Street Institute of Child Health, University College London, UK; i Statistical Laboratory, Centre for Mathematical Sciences, University of Cambridge, UK; j Department of Radiology, University of Cambridge, UK; k Department of Radiology, University Medical Center Groningen, University of Groningen, the Netherlands; l Cancer Trials Unit Department of Oncology, Addenbrooke’s Hospital, Cambridge, UK; m Wolfson Brain Imaging Centre, Department of Clinical Neuroscience, University of Cambridge, UK

**Keywords:** Glioblastoma, Tumor habitat imaging, Heterogeneity, Radioresistance, Perfusion imaging, Diffusion imaging

## Abstract

**Background and purpose:**

Glioblastoma exhibits profound intratumoral heterogeneity in perfusion. Particularly, low perfusion may induce treatment resistance. Thus, imaging approaches that define low perfusion compartments are crucial for clinical management.

**Materials and methods:**

A total of 112 newly diagnosed glioblastoma patients were prospectively recruited for maximal safe resection. The apparent diffusion coefficient (ADC) and relative cerebral blood volume (rCBV) were calculated from diffusion and perfusion imaging, respectively. Based on the overlapping regions of lowest rCBV quartile (rCBV_L_) with the lowest ADC quartile (ADC_L_) and highest ADC quartile (ADC_H_) in each tumor, two low perfusion compartments (ADC_H_-rCBV_L_ and ADC_L_-rCBV_L_) were identified for volumetric analysis. Lactate and macromolecule/lipid levels were determined from multivoxel MR spectroscopic imaging. Progression-free survival (PFS) and overall survival (OS) were analyzed using Kaplan–Meier’s and multivariate Cox regression analyses, to evaluate the effects of compartment volume and lactate level on survival.

**Results:**

Two compartments displayed higher lactate and macromolecule/lipid levels compared to contralateral normal-appearing white matter (each *P <* 0.001). The proportion of the ADC_L_-rCBV_L_ compartment in the contrast-enhancing tumor was associated with a larger infiltration on FLAIR (*P* < 0.001, rho = 0.42). The minimally invasive phenotype displayed a lower proportion of the ADC_L_-rCBV_L_ compartment than the localized (*P* = 0.031) and diffuse phenotypes (not significant). Multivariate Cox regression showed higher lactate level in the ADC_L_-rCBV_L_ compartment was associated with worsened survival (PFS: HR 2.995, *P* = 0.047; OS: HR 4.974, *P* = 0.005).

**Conclusions:**

Our results suggest that the ADC_L_-rCBV_L_ compartment may potentially indicate a clinically measurable resistant compartment.

## Highlights

• Multiparametric MRI may identify two low perfusion compartments in glioblastoma.
• The volume relates to better outcome, while the lactate relates to worse outcome.
• Low perfusion compartment with restricted diffusivity may relates to resistance.

Glioblastoma is a highly aggressive primary brain malignancy in adults [1]. Despite advances in treatment, the median overall survival (OS) of patients remains low at 14.6 months [2]. Inconsistent response is a major challenge in treatment and could be caused by the extensive heterogeneity of this malignancy. Many genetically distinct cell populations can exist in the same tumor and display diverse treatment response [3], [4].

One of the most fundamental traits of glioblastoma is the tumor-related angiogenesis and elevated perfusion, associated with a more invasive phenotype [5]. However, a potent angiogenesis inhibitor failed to demonstrate consistent benefits in clinical trials of *de novo* glioblastoma [6]. One possible explanation is the profound intratumor perfusion heterogeneity in glioblastomas, which is due to aberrant microvasculature and inefficient nutrient delivery. This heterogeneity can give rise to regions within tumors where the demand and supply of nutrients is mismatched [7]. Consequently, the sufficiently perfused habitats may hold the advantages for progression and proliferation, whereas the insufficiently perfused habitats may harbor a more acidic microenvironment than other tumor sub-regions [8], which may preferentially induce resistant clones to adjuvant therapy [9]. There is a rising need to understand the function of low perfusion compartments and evaluate their effects on treatment resistance.

Current clinical practice infers the low perfusion regions as the non-enhancing regions within contrast enhancement on post-contrast images, which can lead to non-specific results using conventional weighted images [10], [11]. Recent studies suggested that quantitative imaging features are useful in reflecting intratumor habitats and tumor microenvironment [12], [13]. As such, multiparametric imaging may allow a more comprehensive evaluation of the low perfusion compartments compared to the morphological heterogeneity visualized by structural magnetic resonance imaging (MRI).

The purpose of this study was to propose a method for quantifying low perfusion compartments in glioblastoma using multiparametric MRI and habitat imaging, and investigate their effects on treatment response and patient outcome. Our hypothesis is that multiparametric MRI may facilitate the identification of clinically relevant intratumor habitats that correlate with patient prognosis.

We integrated perfusion, diffusion and MR spectroscopic imaging with conventional imaging in our study. The relative cerebral blood volume (rCBV) calculated from perfusion imaging measures tumor vascularity [14]. The apparent diffusion coefficient (ADC) calculated from diffusion imaging provides information about tumor microstructure by measuring diffusivity of water molecules [15]. We used a thresholding method to visualize two low perfusion compartments with high/low diffusivity. Thus, two low perfusion compartments, distinguished by two potentially distinct properties, were visualized: one compartment with restricted diffusivity that may represent a sub-region with more microstructure adapting to the acidic conditions [12], and one compartment with increased diffusivity that may represent a sub-region with diminishing microstructure. We studied the metabolic signatures in each compartment using MR spectroscopy. Using multivariate survival analysis, we demonstrated that the volume and lactate level of these two compartments are clinically important.

## Materials and methods

### Patient cohort

Patients with a radiological diagnosis of primary supratentorial glioblastoma suitable for maximal safe surgical resection were prospectively recruited from July 2010 to April 2015. All patients had a good performance status (World Health Organization performance status 0–1). Exclusion criteria included history of previous cranial surgery or radiotherapy/chemotherapy, or inability to undergo MRI scanning. This study was approved by the local institutional review board. Signed informed consent was obtained from all patients.

A total of 131 patients were recruited for the imaging scanning. After surgery, non-glioblastomas were excluded and 112 patients were included for regions of interest (ROI) analysis. Subgroups of patients with available MRS data (58 patients), DTI invasive phenotype data (64 patients) and survival data (80 patients) were analyzed. Patient recruitment and subgroups were summarized in Supplementary Fig. 1.

### Treatment and response evaluation

All patients were on stable doses (8 mg/day) of dexamethasone. Tumor resection was performed with the guidance of neuronavigation (StealthStation, Medtronic) and 5-aminolevulinic acid fluorescence with other adjuvants, e.g., cortical and subcortical mapping, awake surgery, and intraoperative electrophysiology, to allow for maximal safe resection. Extent of resection was assessed according to the postoperative MRI scans within 72 hours as complete resection, partial resection of contrast-enhancing tumor or biopsy [16].

Patients received adjuvant therapy post-operatively according to their performance status. All patients were followed up according to the criteria of response assessment in neuro-oncology (RANO) [17], incorporating clinical and radiological criteria. Survivals were analyzed retrospectively in some cases when pseudoprogression was suspected if new contrast enhancement appeared within first 12 weeks after completing chemoradiotherapy.

### MRI acquisition

All MRI scans were performed at a 3-Tesla MRI system (Magnetron Trio; Siemens Healthcare, Erlangen, Germany) with a standard 12-channel receive-head coil. MRI sequences included: axial T2-weighted, axial T2-weighted fluid-attenuated inversion recovery (FLAIR), diffusion tensor imaging (DTI) with inline ADC calculation, multivoxel 2D ^1^H-MR spectroscopic imaging (MRSI), dynamic susceptibility contrast (DSC) and post-contrast T1-weighted 3D volumetric sequence. Scanning parameters are detailed in Supplementary materials.

### Image processing

For each subject, all images were co-registered to T2-weighted images with an affine transformation, using the linear image registration tool functions [18] in Oxford Centre for Functional MRI of the Brain Software Library (FSL) v5.0.0 (Oxford, UK) [19].

DSC was processed and leakage correction was performed using NordicICE (NordicNeuroLab, Bergen, Norway). The arterial input function was automatically defined and rCBV was calculated. DTI images were processed with the diffusion toolbox in FSL [20], during which normalization and eddy current correction were performed. The isotropic component (p) and anisotropic component (q) were calculated using previous equations [21].

2D ^1^H-MRSI were processed using LCModel (Provencher, Oakville, Ontario) and the concentrations of lactate (Lac) and macromolecule and lipid levels at 0.9 ppm (ML9) were calculated as a ratio to creatine (Cr). The quality and reliability of MRSI, and all spectra were evaluated using previous criteria [22].

### Regions of interest and volumetric analysis

Tumor Regions of interest (ROIs) were manually segmented in 3D slicer v4.6.2 (https://www.slicer.org/) [23] by a neurosurgeon (××, >7 years of experience) and a researcher (××, >4 years of brain tumor image analysis experience) and reviewed by a neuroradiologist (××, >8 years of experience) on post-contrast T1W and FLAIR images using a consistent criteria in each patient, and then cross-validated by comparing the similarity of the delineation using Dice similarity coefficient scores, blinded to the patient outcomes. The contrast-enhancing (CE) ROI was defined as all abnormalities within the contrast-enhancing rim on the post-contrast T1W images. FLAIR ROI was defined as the hyperintense abnormalities on FLAIR images. The interrater variability was tested using Dice similarity coefficient scores. For each individual patient, ROIs of normal-appearing white matter (NAWM) were manually segmented from the contralateral white matter as normal control. This region was typically 10 mm in diameter and located in the white matter which was furthest in distance from the tumor location and has no perceivable abnormalities [24]. All parametric maps of ADC and rCBV were normalized by the mean value in NAWM.

**Fig. 1:**
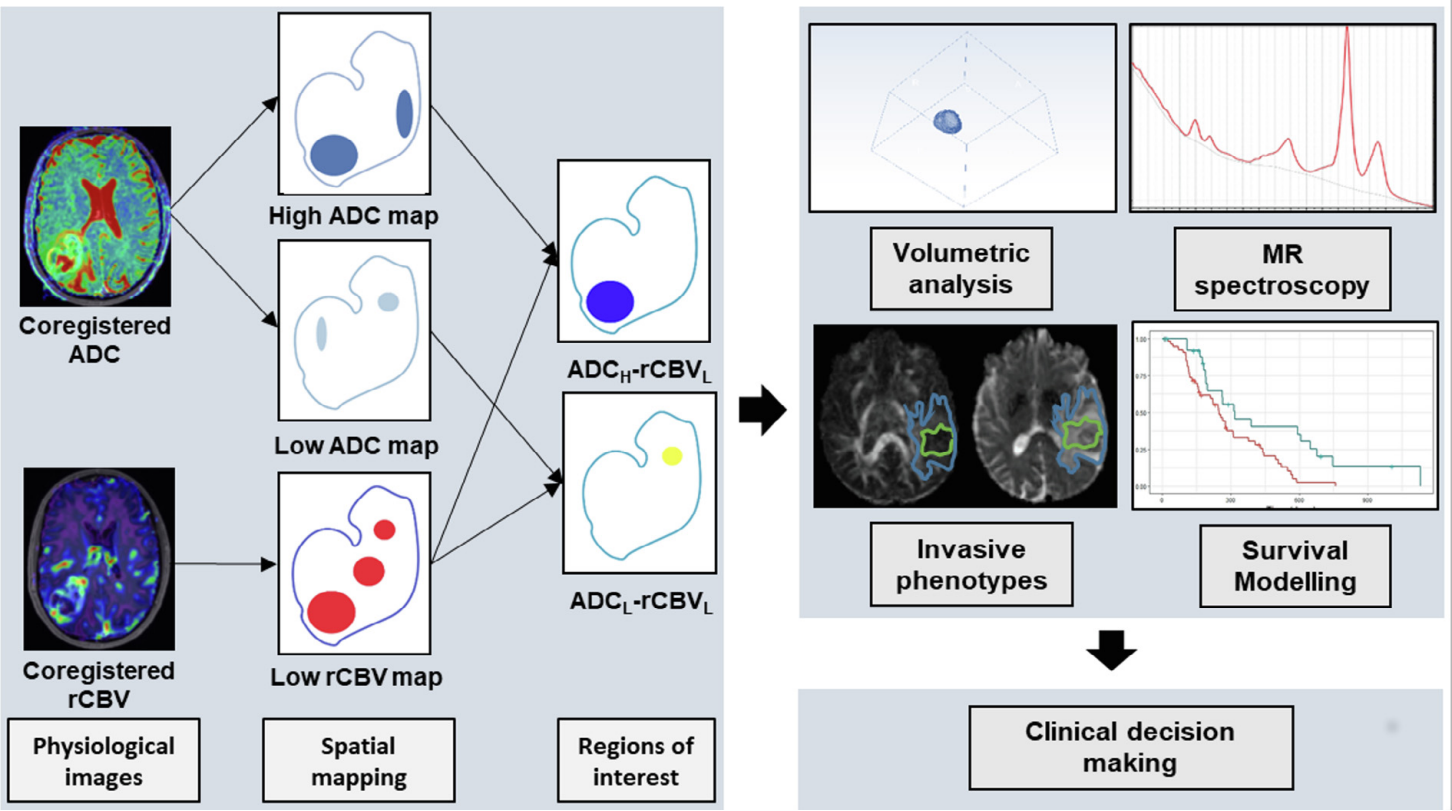
Illustration of the pipeline to identify two ADC-rCBV compartments. Both ADC and rCBV maps are co-registered to the T2 weighted images and tumor regions are segmented manually. Low perfusion regions are partitioned using a quartile threshold. Similarly, two ADC regions are partitioned using high and low ADC quartile thresholds respectively. The spatial overlap between the thresholded rCBV and ADC maps defined two compartments ADC_H_-rCBV_L_ and ADC_L_-rCBV_L_. MR volumetric and metabolic analyses of both compartments are performed and interrogated in invasive phenotype and patient survival analysis models.

ADC-rCBV ROIs were generated using quartile values in MATLAB (v2016a, The MathWorks, Inc., Natick MA) from the CE ROI. The procedure is illustrated in Fig. 1. Firstly, ADC and rCBV values were obtained from each voxel within the CE ROI and pooled together as described previously [24]. The lowest quartile of the pooled rCBV values (rCBV_L_) were interpreted as low perfusion regions. Then the first quartile (ADC_L_) and last quartile (ADC_H_) of ADC map were respectively overlaid on rCBV_L_ maps. Finally, two intersections of ADC_L_-rCBV_L_ and ADC_H_-rCBV_L_ ROIs were obtained. Other regions within CE outside the two ADC-rCBV ROIs were taken as abnormal controls (CE control, CEC). Absolute volumes of ROIs were calculated in FSL [19]. Proportional volumes (%) of two ADC-rCBV ROIs were calculated as the ratio of the absolute volumes to CE volume.

### MRSI voxel selection

Due to the difference in spatial resolution between MRSI and MRI, voxels from T2-weighted MRIs were projected onto MRSI using MATLAB, to evaluate the spectroscopic measure of the ADC-rCBV compartments identified on T2 space. The proportions of the voxels of the ADC-rCBV compartments occupying each MRSI voxel was calculated, and only the MRSI voxels that were completely within the delineated tumor were included in further analyses. Since the ADC-rCBV compartments potentially included multiple MRSI voxels, the proportions of T2-space voxels in the MRSI voxels were calculated and taken as the weight of each MRSI voxel. The sum weighted value was used as the final metabolic value, providing an objective method for MRSI voxel selection (Supplementary Fig. 2).

### DTI invasive phenotypes

We investigated DTI invasive phenotypes of 64 patients which overlap with a previously reported cohort and have been correlated to isocitrate dehydrogenase (IDH) mutation status [25]. Three invasive phenotypes (Supplementary Fig. 3) were classified using previously described criteria [26] based on the decomposition of diffusion tensor into isotropic (p) and anisotropic components (q): (a) diffuse invasive phenotype; (b) localized invasive phenotype; and (c) minimal invasive phenotype.

### Statistical analysis

All analyses were performed with RStudio v3.2.3. Continuous variables were tested with Welch Two Sample t-test. MRSI data or tumor volume, were compared with Wilcoxon’s rank sum test or Kruskal–Wallis’ rank sum test, as appropriate, using Benjamini–Hochberg’s procedure for controlling the false discovery rate in multiple comparisons. Spearman’s rank correlation was used to model the relation between the volume of two ADC-rCBV ROIs and the volume of CE and FLAIR ROIs. Survival was analyzed on patients who received standard treatment following Stupp’s protocol. Kaplan–Meier’s using log-rank test and Cox proportional hazards regression analyses were performed to evaluate patient survival. For Kaplan–Meier’s analysis, the volumes of ROIs and MRSI variables were dichotomized using the ‘surv_cutpoint’ function in R Package “survminer”. Patients who were alive in last follow-up were censored. Multivariate Cox regression with forward and backward stepwise procedures was performed, accounting for relevant covariates, including IDH-1 mutation, MGMT promoter methylation status, sex, age, extent of resection and contrast-enhancing tumor volume. The forward procedure started from the model with one covariate. The backward procedure initiated from the model including all covariates. For each step, Akaike Information Criterion was used to evaluate the model performance. The final multivariate model was constructed using the covariates selected by the stepwise procedures. The hypothesis of no effect was rejected at a two-sided level of 0.05.

## Results

### Patients

The mean age of the 112 patients included was 59.4 years (range 22–76, 84 males; Table 1). Depending on the post-operative status, patients received concurrent temozolomide chemoradiotherapy followed by adjuvant temozolomide following the Stupp protocol (73.2%, 82/112), short-course radiotherapy (17.0%, 19/112), or best supportive care (9.8%, 11/112), respectively. Eighty of 82 (97.6%) patients who received treatment following Stupp’s protocol had data available for survival analysis. The median progression-free survival (PFS) was 265 days (range 25–1130 days) and overall survival was 455 days (range 52–1376 days).

**Table 1:** Patient clinical characteristics and volumes of regions of interest.

Variables	Patient number	CE		FLAIR		ADC_H_-rCBV_L_		ADC_L_-rCBV_L_	
		Mean ± SD (cm^3^)	*P*	Mean ± SD (cm^3^)	*P*	Mean ± SD (cm^3^)	*P*	Mean ± SD (cm^3^)	*P*
*Age at diagnosis*									
<60	38	41.9 ± 24.0	**0.022**	101.4 ± 55.4	0.143	4.3 ± 3.7	**0.020**	1.9 ± 1.6	0.224
≥60	74	58.6 ± 35.7		119.5 ± 63.8		6.3 ± 4.8		2.5 ± 2.4	

*Sex*									
Male	84	54.5 ± 34.1	0.323	115.2 ± 62.8	0.675	5.8 ± 4.6	0.657	2.4 ± 2.3	0.328
Female	28	48.5 ± 30.0		107.7 ± 57.8		5.4 ± 4.4		1.9 ± 1.8	

*Extent of resection*									
Complete	75	45.8 ± 26.0	**0.006**	106.0 ± 58.0	0.062	4.8 ± 3.9	**0.002**	2.1 ± 1.8	0.685
Partial	37	67.4 ± 40.8		128.3 ± 66.1		7.5 ± 5.2		2.6 ± 2.8	

MGMT promoter methylation status*									
Methylated	48	48.4 ± 32.7	0.154	105.4 ± 66.3	0.161	4.8 ± 4.1	0.099	2.2 ± 2.4	0.282
Unmethylated	60	55.8 ± 32.1		121.1 ± 57.9		6.1 ± 4.5		2.4 ± 2.0	

*IDH-1 mutation status*									
Mutant	7	54.6 ± 35.4	0.895	102.2 ± 69.1	0.471	5.8 ± 4.9	0.843	2.2 ± 1.7	0.787
Wild-type	105	52.9 ± 33.1		114.1 ± 61.2		5.6 ± 4.6		2.3 ± 2.2	

Bold values: P < 0.05

* MGMT promoter methylation status unavailable for 4 patients. cm: centimeters; CE: regions including all the abnormalities within contrasting enhancing rim; FLAIR: abnormalities visualized on fluid-attenuated inversion recovery images; ADC_L_-rCBV_L_: overlapping regions of lowest ADC quartile and lowest rCBV quartile; ADC_H_-rCBV_L_: overlapping regions of highest ADC quartile and lowest rCBV quartile; IDH-1: Isocitrate dehydrogenase1; MGMT: O-6-methylguanine-DNA methyltransferase; SD: Standard deviation.

### Multiparametric MRI identified two low perfusion compartments

**Fig. 2:**
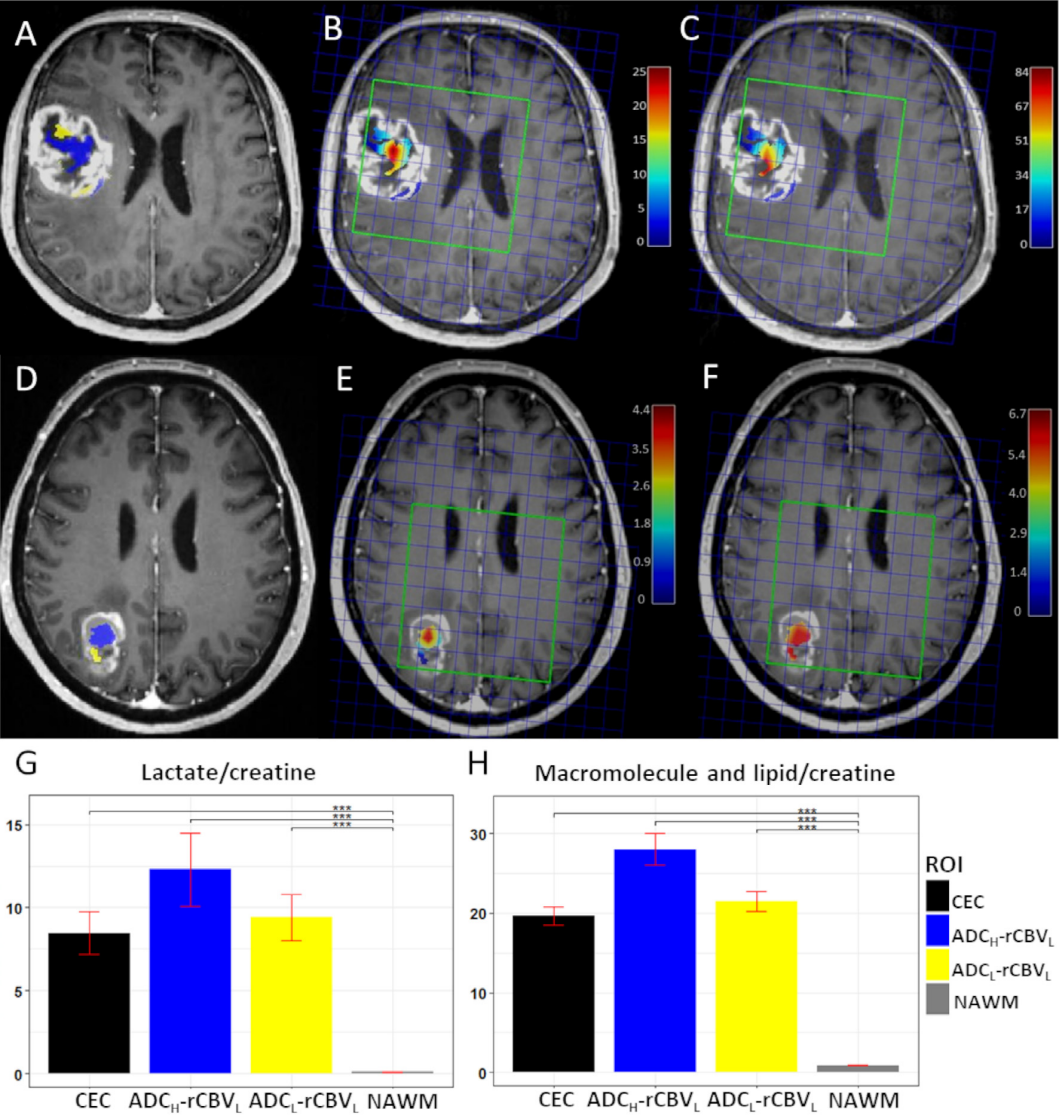
Two hypoxic compartments and MRS characteristics. Case 1: A–C; Case 2: D–F. A & D show the location of ADC_L_-rCBV_L_ (yellow) and ADC_H_-rCBV_L_ (blue) compartments. B & E demonstrate the Lac/Cr ratios of the two compartments. C & F demonstrate the ML9/Cr ratios in the two compartments. The color bar show the level of metabolites (red: high, blue: low). Note that case 1 shows greater tumor volume and higher lactate level. G & H demonstrate the MRSI characteristics of the compartments over the patient cohort. Yellow: ADC_L_-rCBV_L_; blue: ADC_H_-rCBV_L_; black: contrast-enhancing control (CEC); gray: normal-appearing white matter (NAWM). G: mean Lac/Cr level; H: mean ML9/Cr. ^***^P < 0.001. (For interpretation of the references to colour in this figure legend, the reader is referred to the web version of this article.)

The volumes of ROIs for patient subgroups are compared in Table 1. The interrater variability of the ROIs showed excellent agreement, with Dice scores of 0.85 ± 0.10 (CE) and 0.86 ± 0.10 (FLAIR) respectively. The ADC_H_-rCBV_L_ compartment (volume 5.7 ± 4.6 cm^3^) was generally larger than the ADC_L_-rCBV_L_ compartment (volume 2.3 ± 2.2 cm^3^) (*P* < 0.001). Completely resected tumors had smaller CE volume (*P* = 0.006) and smaller ADC_H_-rCBV_L_ compartment (*P* = 0.002). Fig. 2(A, D) shows the two compartments for two cases.

### Low perfusion compartments displayed abnormal metabolic signatures

The ADC_H_-rCBV_L_ compartment showed a significantly higher lactate/creatine (Lac/Cr) ratio than NAWM (*P* < 0.001), and an increased ML9/Cr ratio compared to NAWM (*P* < 0.001). Similarly, the ADC_L_-rCBV_L_ compartment displayed higher Lac/Cr ratio and ML9/Cr ratio than NAWM (both *P* < 0.001). Although not significant, the Lac/Cr and ML9/Cr ratios in the ADC_H_-rCBV_L_ compartment were higher than the ADC_L_-rCBV_L_ compartment (Supplementary Table 1). Fig. 2 shows the comparison of the metabolite levels of two compartments.

### Low perfusion compartments exhibited diverse effects on tumor invasion

The contrast-enhancing (CE) tumor volume was significantly correlated with the Lac/Cr ratio in the ADC_L_-rCBV_L_ (*P* = 0.018, rho = 0.34). Interestingly, the volume of tumor infiltration beyond contrast enhancement, which was delineated on FLAIR images and normalized by CE volume, showed a moderate positive correlation with the proportional volume of the ADC_L_-rCBV_L_ compartment (*P* < 0.001, rho = 0.42) and a negative correlation with the proportional volume of the ADC_H_-rCBV_L_ compartment (*P* < 0.001, rho = −0.32). The correlations of ROI volumes are demonstrated in Supplementary Fig. 4.

### The ADC_L_-rCBV_L_ compartment of minimally invasive tumors showed lower lactate

The minimally invasive phenotype displayed a lower proportional volume of ADC_L_-rCBV_L_ compartment in CE ROI than the localized (*P* = 0.031) and diffuse phenotype (not significant), and a higher proportion of ADC_H_-rCBV_L_ compartment than the localized (*P* = 0.024) and diffuse phenotype (not significant), suggesting the effects of the two low perfusion compartments to tumor invasiveness were different. Of note, the minimally invasive phenotype displayed lower Lac/Cr ratio compared to the localized (*P* = 0.027) and diffuse phenotype (*P* = 0.044), indicating that the ADC_L_-rCBV_L_ compartment may have less acidic microenvironment in the minimally invasive tumors. A full comparison between the three invasive phenotypes can be found in Supplementary Table 2.

**Table 2:** Multivariate and Stepwise modeling of patient survivals.

Factors	PFS						OS					
	Multivariate			Stepwise			Multivariate			Stepwise		
	HR	95%CI	*P*	HR	95%CI	*P*	HR	95%CI	*P*	HR	95%CI	*P*
Age	1.007	0.979–1.035	0.645				1.002	0.971–1.033	0.911	0.927	0.860–0.998	**0.045**
Sex (M)	1.499	0.838–2.681	0.172	5.043	1.063–23.91	**0.042**	1.252	0.662–2.365	0.490			
EOR (partial resection)	2.825	1.417–5.635	**0.003**	4.531	1.002–20.49	**0.050**	2.063	1.099–3.874	**0.024**	12.18	2.701–54.91	**0.001**
MGMT promoter methylation status*	0.624	0.366–1.063	0.083	0.392	0.125–1.233	0.109	0.647	0.358–1.167	0.148	0.231	0.070–0.762	**0.016**
IDH-1 mutation status	0.902	0.278–2.926	0.864				0.900	0.256–3.170	0.870			
CE volume	1.291	0.861–1.935	0.216	6.760	0.696–65.63	0.099	2.311	1.527–3.499	**<0.001**	3.080	1.487–6.383	**0.002**
FLAIR volume	0.775	0.519–1.157	0.212	2.008	0.818–4.926	0.128	0.653	0.444–0.961	**0.031**			
ADC_L_-rCBV_L_ volume				0.184	0.039–0.874	**0.033**						
ADC_H_-rCBV_L_ volume				0.102	0.011–0.992	**0.049**						
Lac/Cr in ADC_H_-rCBV_L_				6.562	2.023–21.29	**0.002**				2.367	0.825–6.790	0.109
Lac/Cr in ADC_L_-rCBV_L_				2.995	1.012–8.861	**0.047**				4.974	1.608–15.39	**0.005**
Lac/Cr in CEC				0.053	0.010–0.295	**0.001**				0.090	0.016–0.520	**0.007**

Bold values: P < 0.05

* MGMT-methylation status unavailable for 4 patients. EOR: extent of resection. PFS: progression-free survival; OS: overall survival; HR: hazard ratio; CI: confidence interval; IDH-1: Isocitrate dehydrogenase1; MGMT: O-6-methylguanine-DNA methyltransferase; CE: regions including all the abnormalities within contrasting enhancing rim; FLAIR: abnormalities visualized on fluid-attenuated inversion recovery images; Lac: lactate; Cr: creatine; ADC_L_-rCBV_L_: overlapping regions of lowest ADC quartile of and lowest rCBV quartile; ADC_H_-rCBV_L_: overlapping regions of highest ADC quartile and lowest rCBV quartile; CEC: contrast enhanced control.

### Low perfusion compartments exhibited diversity in treatment response

First, we used multivariate Cox regression to analyze all relevant clinical covariates. The results showed that extent of resection (EOR) (PFS: hazard ratio [HR] = 2.825, *P* = 0.003; OS: HR = 2.063, *P* = 0.024), CE tumor volume (OS: HR = 2.311, *P* < 0.001) and FLAIR tumor volume (OS: HR = 0.653, *P* = 0.031) were significantly associated with survivals.

**Fig. 3:**
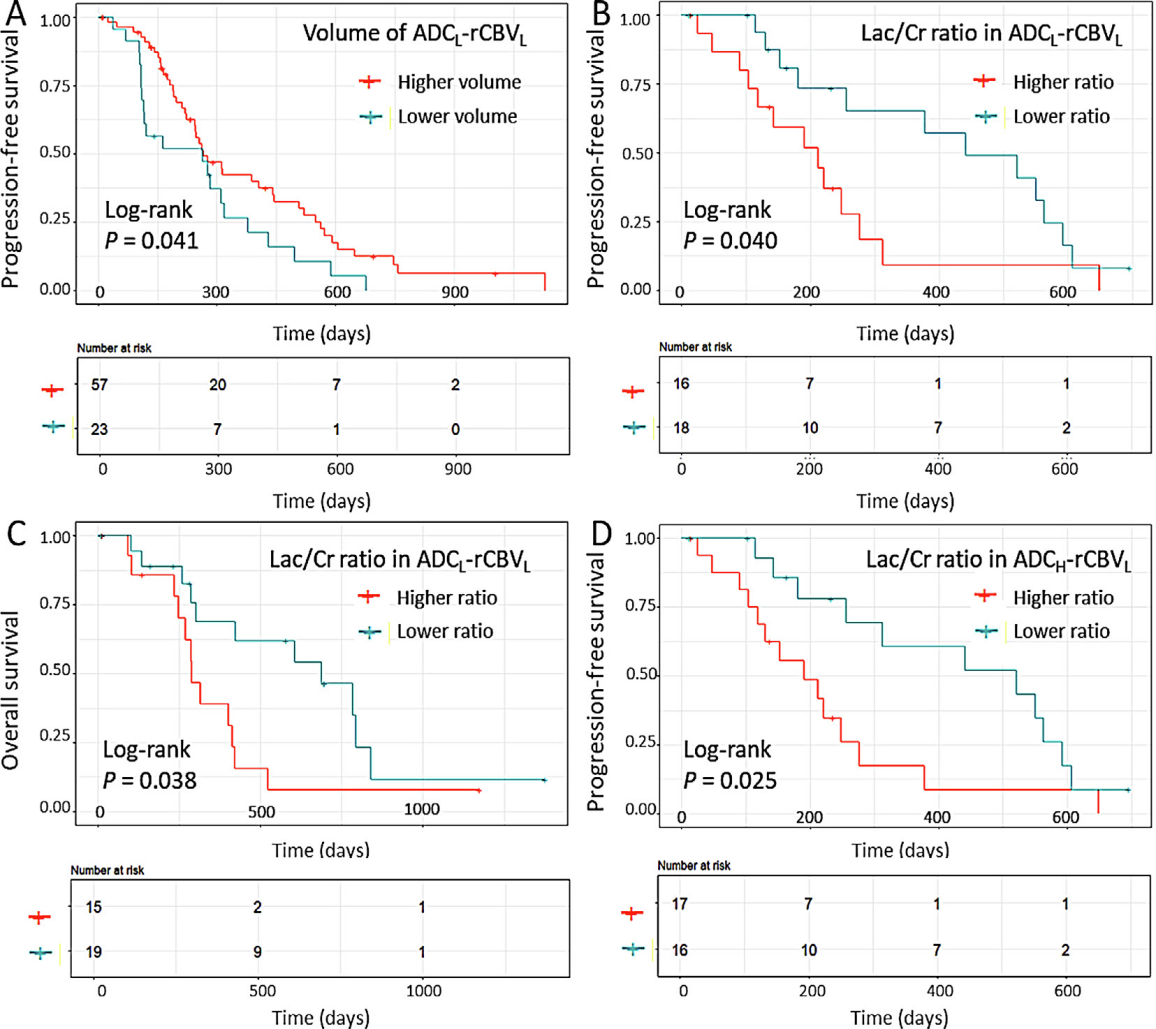
Kaplan–Meier’s plots of survival analysis. Log-rank tests show larger proportional volume of ADC_L_-rCBV_L_ compartment is associated with better PFS (*P* = 0.041) (A), while higher Lac/Cr ratio in this compartment is associated with worse PFS (*P* = 0.040) (B) and OS (*P* = 0.038) (C). Higher Lac/Cr ratio in the ADC_H_-rCBV_L_ compartment is associated with worse PFS (*P* = 0.025) (D).

Next, we included the volumes of two compartments and their Lac/Cr ratios into the survival models. The results using a stepwise procedure showed that higher volumes of the two compartments were associated with better PFS (ADC_H_-rCBV_L_: HR = 0.102, *P* = 0.049; ADC_L_-rCBV_L_: HR = 0.184, *P* = 0.033), while the higher Lac/Cr ratio in the two compartments was associated with worse PFS (ADC_H_-rCBV_L_: HR = 6.562, *P* = 0.002; ADC_L_-rCBV_L_: HR = 2.995, *P* = 0.047). Further, the higher Lac/Cr ratio in the ADC_L_-rCBV_L_ compartment was also associated with worse OS (HR = 4.974, *P* = 0.005). In contrast, the Lac/Cr ratio in the contrast-enhancing control regions was associated with better survivals (PFS: HR = 0.053, *P* = 0.001; OS: HR = 0.090, *P* = 0.007). The results of the Cox proportional hazards models are described in Table 2 and the Kaplan–Meier curves using log-rank test are shown in Fig. 3.

## Discussion

This study combined perfusion and diffusion parameters to quantify the low perfusion compartments that may be responsible for treatment resistance. The non-invasive approach using physiological imaging may potentially improve the commonly used weighted structural imaging.

The clinical values of the individual markers have been assessed previously. Among them, rCBV is reported to indicate IDH mutation status and associated with hypoxia-initiated angiogenesis [27]. Decreased ADC reflects restricted diffusivity, which is considered to represent higher tumor cellularity/cell packing [28] and associated with shorter survival [29]. Of note, although another meta-analysis also showed that the ADC value had an inverse correlation with cellularity in glioma, this correlation was not consistent in all tumor types [30]. Here we integrated multiparametric MRI to identify two low perfusion compartments. With similar low levels of perfusion, the restricted diffusivity in the ADC_L_-rCBV_L_ compartment suggests this compartment may contain more microstructures, compared to the ADC_H_-rCBV_L_ compartment.

We measured the lactate, macromolecule and lipid levels at 0.9 ppm (ML9) in the spectra, as increased lactate indicates an acidic microenvironment, while ML9 is associated with pro-inflammatory microglial response [31]. The elevated ML9/Cr ratios may suggest both compartments displayed elevated inflammation response [31], potentially due to recruitment of inflammatory cells by necrotic tissue [32]. The positive correlation between tumor volume and lactate levels in the ADC_L_-rCBV_L_ compartment could indicate a higher lactate production as tumor grows. When evaluating the non-enhancing peritumoral regions, we found that tumors with larger infiltration area tended to have smaller ADC_H_-rCBV_L_ and larger ADC_L_-rCBV_L_ compartments, suggesting the latter might be more responsible for infiltration. This was supported by our findings that minimally invasive phenotypes displayed significantly lower lactate in the ADC_L_-rCBV_L_ compartment.

We further investigated the effects of two compartments on patient survivals. Interestingly, a higher Lac/Cr ratio in the two compartments was related to elevated hazard (HR > 1) while this ratio in other tumor regions showed a reduced hazard. This implies that the resistant phenotype may possibly reside in the two compartments. As the ADC_L_-rCBV_L_ compartment was associated with larger tumor infiltration area, higher diffusion invasiveness, and the lactate level in this compartment had significant effect on both PFS and OS, this compartment may be associated with treatment resistance.

We found that higher volumes of both low perfusion compartments were associated with better survivals, while higher Lac/Cr ratios of these compartments were associated with worse survivals. These results suggested the higher proportion of the low perfusion compartments may indicate relatively lower tumor proliferation; the higher levels of lactate production in these compartments, however, may indicate a more aggressive phenotype. Consistent with our findings, previous studies showed that higher levels of lactate production were associated with radioresistance [33] and worse patient survival [34], potentially due to the antioxidative capacity of lactate.

Our findings have clinical significance. The identification of possibly resistant compartments could inform choice of treatment target. Our results show that higher acidic stress in ADC_L_-rCBV_L_ compartment may lead to a more aggressive phenotype. Since adjuvant therapies may aggravate the microenvironmental stress, this finding suggests that more attention may be needed for patients with larger volumes of ADC_L_-rCBV_L_ compartment.

There are limitations in our study. Firstly, due to the low spatial resolution of MRSI, the multivariate analysis was based on a subset of patients. The low spatial resolution of MRSI can also affect the comparison of the metabolic signatures of the two ADC-rCBV compartments. However, we have used a weighting method to include multiple MRSI voxels containing the two compartments, which may potentially help to reduce this bias caused by a single voxel containing both compartments. Secondly, the cut-off values defining the two compartments were based on the quartiles of rCBV and ADC distributions in individual lesion. We hypothesized that each individual tumor can be an independent ecological system in which the selective stress may arise from the disparities in sub-regional perfusion and diffusion. Compared to our method, a global absolute threshold across all patients may have the advantage of identifying consistent tumor habitats among patients. Several limitations, however, may still potentially exist when using the absolute threshold, even if we have normalized all the pixel values to the contralateral normal-appearing whiter matter. It may not address our hypothesis of intratumoral habitats with evolutionary disadvantages. More importantly, it could be significantly affected by the profound tumor heterogeneity and limited by the scanning setting used in this specific cohort. Thirdly, when the sequences of this study were designed, there was still no consensus regarding the use of pre-bolus of contrast agent. Therefore, a pre-bolus was not given in this study. However, to address this issue, we have used NordicICE to perform leakage correction across all patients using the same software setting. Lastly, although the imaging markers are validated histologically from other studies [35], a full biological validation can only be achieved with multi-region sampling of each tumor.

In conclusion, we showed that multiparametric habitat imaging could identify two low perfusion compartments, which may help improve the non-specific conventional imaging. The compartment demonstrating both low perfusion and restricted diffusion may indicate a habitat especially responsible for treatment resistance. This could provide crucial information for personalized treatment. As our analyses were based on clinically available imaging modalities, this approach could easily be implemented, and potentially extended to other system.

## Conflict of interest

None.

## Funding

The research was supported by the National Institute for Health Research (NIHR) Brain Injury MedTech Co-operative based at Cambridge University Hospitals NHS Foundation Trust and University of Cambridge. The views expressed are those of the author(s) and not necessarily those of the NHS, the NIHR or the Department of Health and Social Care (SJP, project reference NIHR/CS/009/011); CRUK core grant C14303/A17197 and A19274 (FM lab); Cambridge Trust and China Scholarship Council (CL & SW); the Chang Gung Medical Foundation and Chang Gung Memorial Hospital, Keelung, Taiwan (JLY); CRUK & EPSRC Cancer Imaging Centre in Cambridge & Manchester (FM & TT, grant C197/A16465); NIHR Cambridge Biomedical Research Centre (TM & SJP).
