# Supplementary material for: Low perfusion compartments in glioblastoma quantified by advanced magnetic resonance imaging and correlated with patient survival
Source: Radiother Oncol. 2019 May;134:17–24. doi: 10.1016/j.radonc.2019.01.008 (PMC6486398; doi:10.1016/j.radonc.2019.01.008)
Supplement: Supplementary data 1 [file mmc1.docx]

**Pre-operative MRI acquisition**

MRI sequences were acquired as following: T2-weighted sequence (TR/TE 4840-5470/114 ms; refocusing pulse flip angle 150°; FOV 220 × 165 mm; 23-26 slices; 0.5 mm slice gap; voxel size of 0.7 × 0.7 × 5.0 mm); T2-weighted fluid attenuated inversion recovery (FLAIR) (TR/TE/TI 7840-8420/95/2500 ms; refocusing pulse flip angle 150°; FOV 250 × 200 mm; 27 slices; 1 mm slice gap; voxel size of 0.78125 × 0.78125 × 4.0 mm). DTI was acquired before contrast enhanced imaging, with a single-shot echo-planar sequence (TR/TE 8300/98 ms; flip angle 90°; FOV 192 × 192 mm; 63 slices; no slice gap; voxel size 2.0 × 2.0 × 2.0 mm; 12 directions; b values: 350, 650, 1000, 1300, and 1600 sec/mm^2^; imaging time: 9 minutes 26 seconds). An inline ADC calculation was performed during DTI acquisition from the scanner using b values of 0–1000 sec/mm^2^. Multivoxel 2D ^1^H-MRS chemical shift imaging utilized a semi-LASER sequence (TR/TE 2000/30-35 ms; flip angle 90°; FOV 160 × 160 mm; voxel size 10 × 10 × 15-20 mm). PRESS excitation was selected to encompass a grid of 8 rows × 8 columns on T2-weighted images. Perfusion weighted imaging was acquired with a dynamic susceptibility contrast (DSC) sequence (TR/TE 1500/30 ms; flip angle 90°; FOV 192 × 192 mm; FOV 192 × 192 mm; 19 slices; slice gap 1.5 mm; voxel size of 2.0 × 2.0 × 5.0 mm; acquisition time 141 seconds) with 9 mL gadobutrol (Gadovist,1.0 mmol/mL; Bayer, Leverkusen, Germany) followed by a 20 mL saline flush administered via a power injector at 5 mL/s. Post-contrast T1-weighted sequence (TR/TE/TI 2300/2.98/900 ms; flip angle 9°; FOV 256 × 240 mm; 176-208 slices; no slice gap; voxel size 1.0 × 1.0 × 1.0 mm) was acquired after intravenous injection of 9 mL gadobutrol.


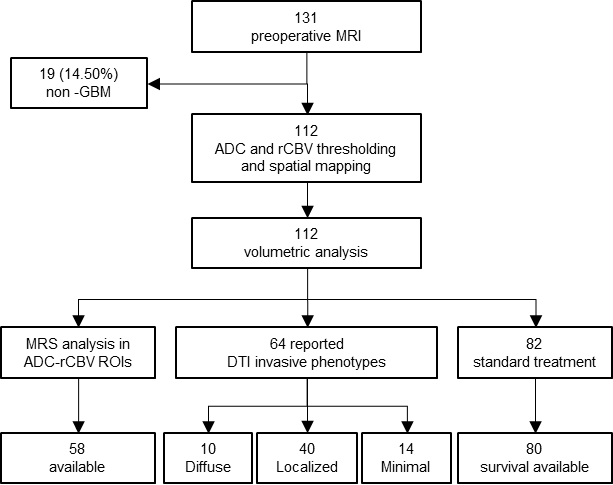


**Supplementary Figure 1. Flow diagram of study design and patient recruitment.** Nineteen patients were excluded due to pathological non-glioblastoma diagnosis. Due to the criteria of multiple voxel selection, patients with missing Lac/Cr and ML9/Cr data were excluded in MRS analysis. DTI invasive phenotypes were correlated with the 64 patients overlapping with a previously reported cohort. Patient survival was reviewed retrospectively to exclude psuedoprogression and was only analyzed in those who received standard chemoradiotherapy.


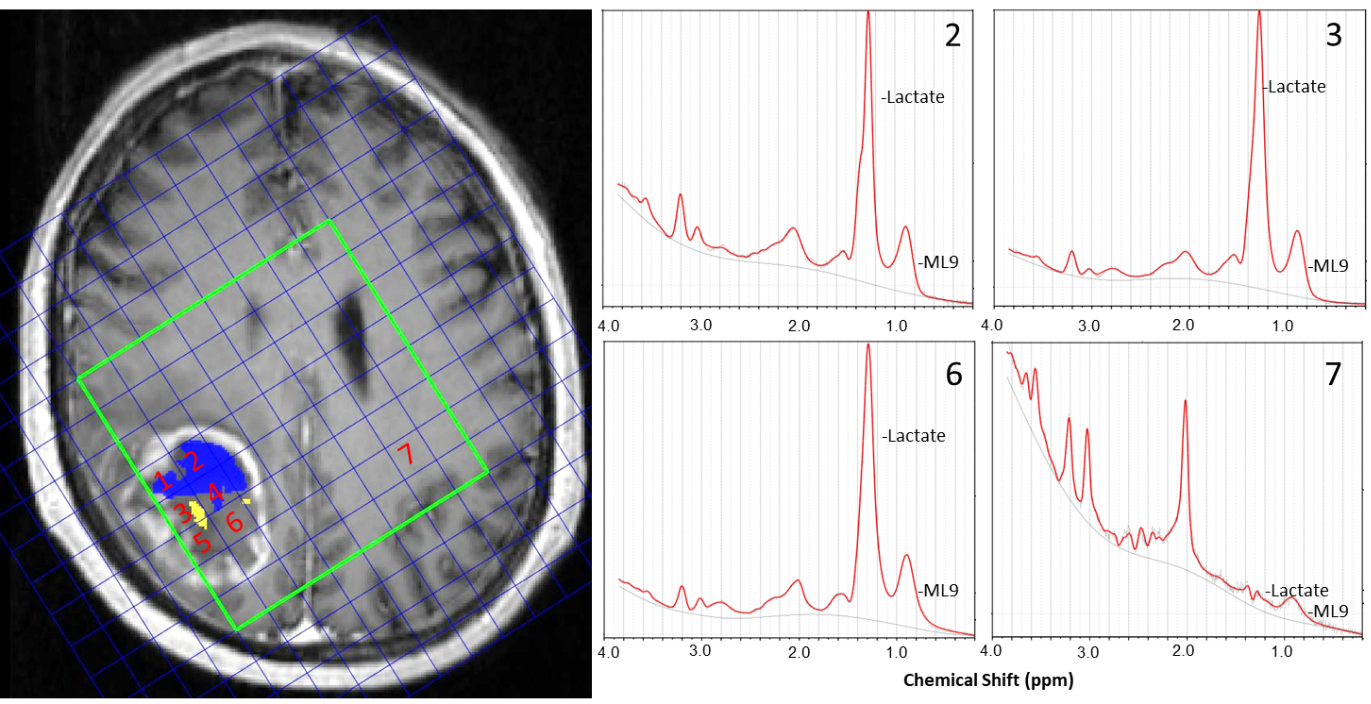


**Supplementary Figure 2. Illustration of multiple voxel MRS analysis**. Left: the selection criteria. The T2-space pixels are projected to MRS space according to their coordinates. The proportion of T2-space tumor pixels occupying each MRS voxel is calculated. A criterion is applied that only those MRS voxels are included when this voxel is completely within delineated tumor. In this case, grid 1-6 met the criteria. A weighted average metabolite content for each region is calculated from the metabolite content of each voxel within it weighted by that voxel’s percentage of (ADC_H_-rCBV_L_ [blue]: grid 1,2 and 4 are counted; ADC_L_-rCBV_L_ [yellow], grid 3 and 5 are counted; abnormal control [CEC]: grid 1-6 are counted). Right: Example spectra of ROIs. Each spectrum corresponds to the grids on the left. Grid 2: lactate/Cr ratio 13.2, ML9/Cr ratio: 10.4; grid 3: lactate/Cr ratio 28.4, ML9/Cr ratio: 22.7; grid 6: lactate/Cr ratio 8.9, ML9/Cr ratio: 16.6; grid 7 (NAWM): lactate/Cr ratio 0.37, ML9:1.36.


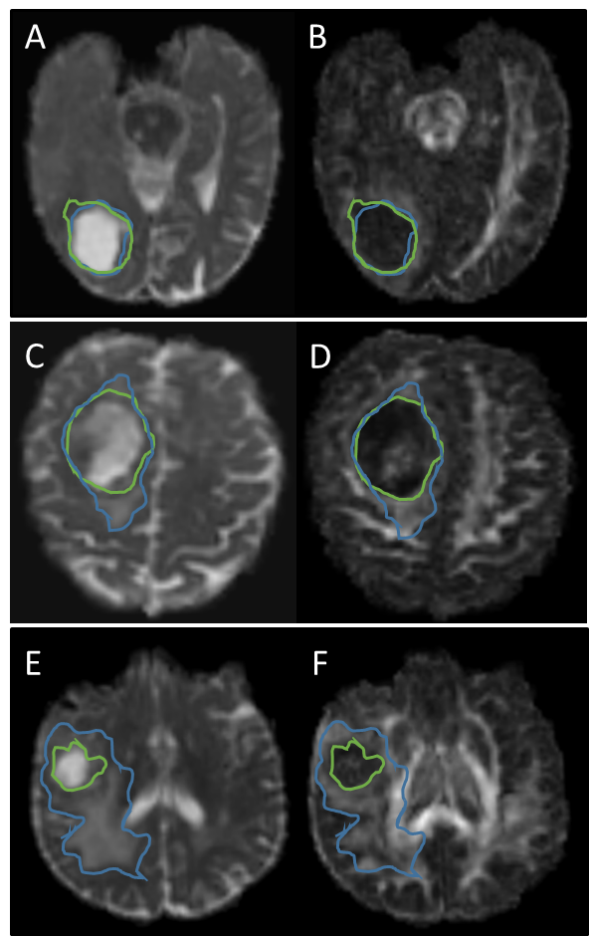


**Supplementary Figure 3. Examples of DTI invasive phenotypes.** (A), (C), (E): DTI-p maps with abnormality outlined by the blue line; (B), (D), (F): DTI-q maps with abnormality outlined by the green line. (A) & (B) show a minimal invasive phenotype. The isotropic abnormality is similar to the anisotropic abnormality. (C) & (D) show a localized invasive phenotype. The isotropic abnormality is larger than the anisotropic abnormality in one direction. (E) & (F) show a diffuse invasive phenotype. The isotropic abnormality is larger than the anisotropic abnormality in more than one direction.


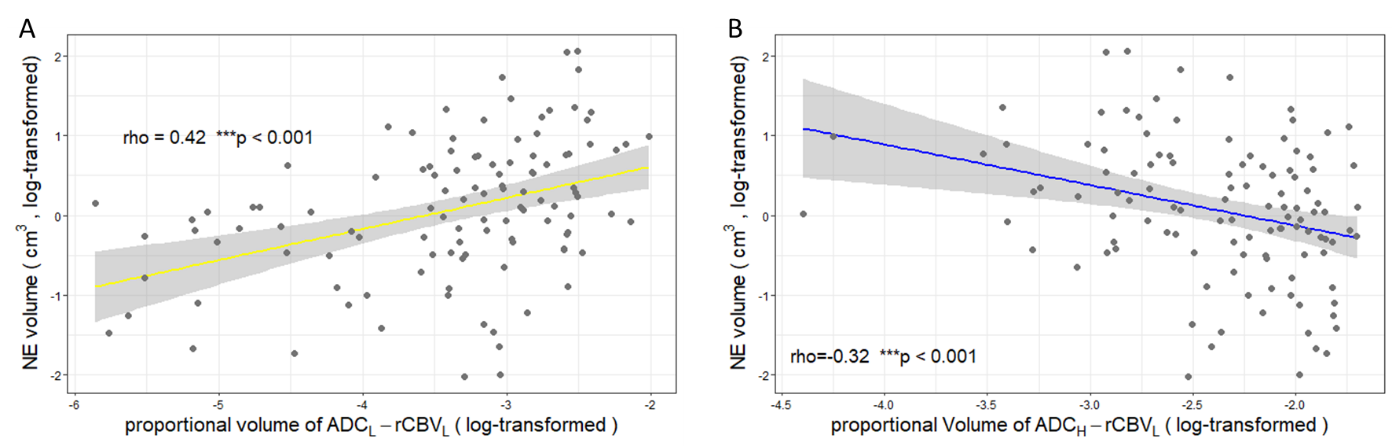


**Supplementary Figure 4. Correlations between two compartments with contrast enhanced tumor and tumor infiltration**. Non-enhancing (NE) tumor (measured by the volume of FLAIR beyond contrast enhancement and normalized by the contrast-enhancing tumor volume) showed a moderate positive correlation with the proportional volume (log-transformed percentage in the contrast-enhancing tumor volume) of the ADC_L_-rCBV_L_ compartment (A) and negative correlation with the proportional volume (log-transformed) of the ADC_H_-rCBV_L_ compartment (B). ***: *P* < 0.001.

**Supplementary Table 1: MRSI characteristics of regions of interest**

| Lac/Cr | | | | | |
| --- | --- | --- | --- | --- | --- |
|  | Descriptive | | ADC_L_-rCBV_L_ | CEC | NAWM |
| ROI | Mean ± SD | 95% CI | *P* | *P* | *P* |
| ADC_H_-rCBV_L_ | 12.3±15.4 | 7.9-16.7 | 0.427 | 0.411 | < 0.001 |
| ADC_L_-rCBV_L_ | 9.4±9.8 | 6.6-12.2 | / | 0.407 | < 0.001 |
| CEC | 8.5±8.9 | 5.9-11.0 | / | / | < 0.001 |
| NAWM | 0.10±0.28 | 0.02-0.17 | / | / | / |
| ML9/Cr | | | | | |
|  | Descriptive | | ADC_L_-rCBV_L_ | CEC | NAWM |
| ROI | Mean ± SD | 95% CI | *P* | *P* | *P* |
| ADC_H_-rCBV_L_ | 28.0±71.6 | 9.2-46.9 | 0.650 | 0.529 | < 0.001 |
| ADC_L_-rCBV_L_ | 21.5±31.2 | 13.3-29.7 | / | 0.492 | < 0.001 |
| CEC | 19.7±29.3 | 12.0-27.4 | / | / | < 0.001 |
| NAWM | 0.87±0.68 | 0.70-1.05 | / | / | / |
| ROI: region of interest; ADC_L_-rCBV_L_: overlapping regions of lowest ADC quartile and lowest rCBV quartile; ADC_H_-rCBV_L_: overlapping regions of highest ADC quartile and lowest rCBV quartile; CEC: contrast enhancement control; NAWM: normal appearing white matter; Lac: lactate; ML9: Macromolecule and lipid at 0.9ppm; Cr: creatine; CI: confidence interval. | | | | | |

**Supplementary Table 2: Comparisons of the volumes and lactate of three DTI invasive phenotypes**

|  | | Diffuse | Localised | Minimal | Comparisons | | |
| --- | --- | --- | --- | --- | --- | --- | --- |
|  | | 10 (15.6%) | 40 (62.5%) | 14 (21.9%) | Localised-Diffuse | Minimal- Diffuse | Minimal-Localised |
| ROI | Variable | Mean ± SD | Mean ± SD | Mean ± SD | *P* | *P* | *P* |
| CE | Volume (cm^3^) | 53.8±35.0 | 39.7±16.0 | 52.7±36.2 | 0.388 | 0.406 | 0.390 |
| FLAIR | Volume (cm^3^) | 116.7±57.3 | 82.0±31.5 | 85.2±68.4 | 0.067 | 0.062 | 0.490 |
| ADC_H_-rCBV_L_ | Volume^#^ | 0.10±0.04 | 0.08±0.03 | 0.12±0.04 | **0.036** | 0.140 | **0.024** |
| ADC_L_-rCBV_L_ | Volume^#^ | 0.04±0.03 | 0.06±0.03 | 0.03±0.02 | 0.080 | 0.104 | **0.031** |
| ADC_H_-rCBV_L_ | Lac/Cr | 12.9±20.3 | 12.9±12.3 | 3.5±3.8 | 0.206 | 0.182 | 0.190 |
| ADC_L_-rCBV_L_ | Lac/Cr | 9.0±9.1 | 10.0±8.9 | 1.8±2.3 | 0.407 | **0.044** | **0.027** |
| CEC | Lac/Cr | 8.8±9.2 | 8.8±7.6 | 2.7±2.7 | 0.218 | 0.080 | 0.073 |
| ^*^absolute volumes; ^#^proportional volumes; ROI: region of interest; CE: contrast enhancement; cm: centimeters; FLAIR: fluid attenuated inversion recovery; ADC_L_-rCBV_L_: overlapping regions of lowest ADC quartile and lowest rCBV quartile; ADC_H_-rCBV_L_: overlapping regions of highest ADC quartile and lowest rCBV quartile; CEC: contrast enhancement control; Lac: lactate; Cr : creatine ; SD: Standard deviation. | | | | | | | |
